# Safety and Efficacy of Magnetic Resonance-Guided Focused Ultrasound Surgery With Autofocusing Echo Imaging

**DOI:** 10.3389/fnins.2020.592763

**Published:** 2021-01-12

**Authors:** Kyung Won Chang, Itay Rachmilevitch, Won Seok Chang, Hyun Ho Jung, Eyal Zadicario, Oleg Prus, Jin Woo Chang

**Affiliations:** ^1^Department of Neurosurgery, Brain Research Institute, Yonsei University College of Medicine, Seoul, South Korea; ^2^InSightec^®^ Ltd., Haifa, Israel

**Keywords:** essential tremor, Parkinson’s disease, auto-focusing, echo imaging, magnetic resonance-guided focused ultrasound surgery

## Abstract

**Objective:**

Magnetic resonance-guided focused ultrasound surgery (MRgFUS) lesioning is a new treatment for brain disorders. However, the skull is a major barrier of ultrasound sonication in MRgFUS because it has an irregular surface and varies its size and shape among individuals. We recently developed the concept of skull density ratio (SDR) to select candidates for MRgFUS from among patients with essential tremor (ET). However, SDR is not the only factor contributing to successful MRgFUS lesioning treatment—refining the target through exact measurement of the ultrasonic echo in the transducer also improves treatment efficacy. In the present study, we carried out MRgFUS lesioning using an autofocusing echo imaging technique. We aimed to evaluate the safety and efficacy of this new approach, especially in patients with low SDR in whom previous focusing methods have failed.

**Methods:**

From December 2019 to March 2020, we recruited 10 patients with ET or Parkinson’s disease (PD) who had a low SDR. Two patients dropped out of the trial due to the screening failure of other medical diseases. In total, eight patients were included: six with ET who underwent MRgFUS thalamotomy and two with PD who underwent MRgFUS pallidotomy. The autofocusing echo imaging technique was used in all cases.

**Results:**

The mean SDR of the patients with ET was 0.34 (range: 0.29–0.39), while that of the patients with PD was 0.41 (range: 0.38–0.44). The mean skull volume of patients with ET was 280.57 cm^3^ (range: 227–319 cm^3^), while that of the patients with PD was 287.13 cm^3^ (range: 271–303 cm^3^). During MRgFUS, a mean of 15 sonications were performed, among which a mean of 5.63 used the autofocusing technique. The mean maximal temperature (Tmax) achieved was 55.88°C (range: 52–59°C), while the mean energy delivered was 34.75 kJ (range: 20–42 kJ) among all patients. No serious adverse events occurred during or after treatment. Tmax or sonication factors (skull volume, SDR, sonication number, autofocusing score, similarity score, energy range, and power) were not correlated with autofocusing technique (*p* > 0.05, autofocusing score showed a *p*-value of 0.071).

**Conclusion:**

Using autofocusing echo imaging lesioning, a safe and efficient MRgFUS treatment, is available even for patients with a low SDR. Therefore, the indications for MRgFUS lesioning could be expanded to include patients with ET who have an SDR < 0.4 and those with PD who have an SDR < 0.45.

**Clinical Trial Registration:**

clinicaltrials.gov, identifier: NCT03935581.

## Introduction

Magnetic resonance-guided focused ultrasound surgery (MRgFUS) through the human skull is a novel treatment for functional brain disorders. It has been widely applied to treat movement disorders such as essential tremor (ET; Chang et al., 2015, 2016, 2019; Elias et al., 2016; Gallay et al., 2016; Chazen et al., 2018; Jung et al., 2018; Wang et al., 2018; Gasca-Salas et al., 2019) and Parkinson’s disease (PD; Na et al., 2015; Bond et al., 2017; Weintraub and Elias, 2017; Martinez-Fernandez et al., 2018), intractable neuropathic pain, and even neuropsychiatric disorders such as obsessive–compulsive disorder (Jeanmonod et al., 2012; Jung et al., 2015; Chang et al., 2016; Kim et al., 2018). It is a relatively safe procedure with minimal side effects and proven efficacy in various diseases (Na et al., 2015; Bond et al., 2017; Chazen et al., 2018; Wang et al., 2018; Gasca-Salas et al., 2019).

However, MRgFUS has some limits. The skull is barely penetrable to ultrasound because bone has a high absorption of ultrasonic energy. Therefore, in some patients, MRgFUS cannot reach the therapeutic temperature of over 54°C necessary for ablative brain lesions (Wang et al., 2018; Chang et al., 2019; D’Souza et al., 2019; Jung et al., 2019). To address this, we developed the concept of skull density radio (SDR) as a factor to predict such treatment failure (Chang et al., 2016). The SDR reflects the uniformity of skull density, which heavily affects the penetration properties of the skull. An SDR below 0.4 (low SDR) is generally considered inadequate or inconducive to optimal thermal lesioning in MRgFUS. Until recently, SDR was the only factor known to influence maximum temperature (Tmax) with relative low sonication energy (Chang et al., 2016, 2019; Wang et al., 2018; Boutet et al., 2019; D’Souza et al., 2019; Jung et al., 2019). However, other factors contributing to successful lesioning in MRgFUS treatment have now been discovered (Chang et al., 2016, 2019; Wang et al., 2018; Boutet et al., 2019; D’Souza et al., 2019; Jung et al., 2019).

In addition, ultrasonic waves passing through the skull are heavily distorted. Although the skull is round, it has an irregular surface and varies in size and shape among individuals. As a result, its focal point, global thickness, and SDR also vary. Moreover, different therapeutic targets, such as the pallidum and the more lateral thalamus, require different incident angles. Therefore, focusing on different targets and modifying the incident angle using the transducer can also affect treatment outcomes (Jung et al., 2019). Hence, an effective focusing technique is essential for treatment success.

The Insightec Exablate 4000 MRgFUS system uses a computed tomography (CT)-based acoustic model of the patient’s skull as a focusing guide. This model simulates ultrasound phase distortion and correction *in situ* during clinical treatment. This approach is successful in high-SDR skulls when the target is near the geometrical center of the skull, such as in ET treatments in the ventral intermediate nucleus (VIM; Chang et al., 2016). When the target is located further from the skull center (typically ≥ 20 mm) and in the case of a low SDR, the likelihood of lesion induction and treatment success drops significantly (Jung et al., 2019).

In the present clinical study, we performed MRgFUS using the new autofocusing echo imaging technique—an Insightec-developed method that uses acoustic signals from microbubbles of ultrasound contrast agent administered intravenously and is activated when the ultrasound focuses on the target area in patients with ET or PD undergoing MRgFUS thalamotomy or pallidotomy. We aimed to evaluate the safety and efficacy of this new approach, especially in patients with low SDR in whom previous focusing methods have failed.

## Methods

**FIGURE 1:**
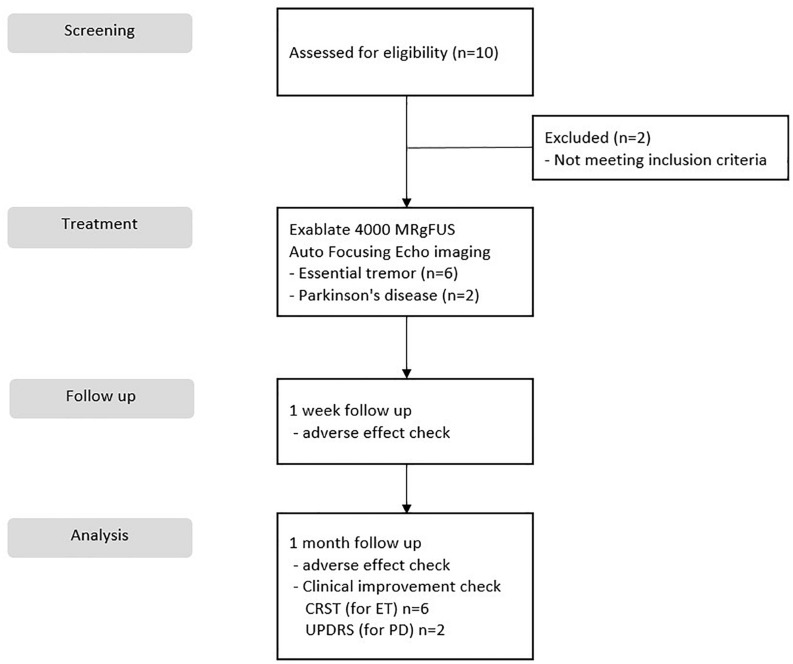
CONSORT flow diagram of patients for the study.

This study was a prospective, single-center, open label, and clinical trial. Data from the autofocusing echo imaging technique were collected at Yonsei University College of Medicine, Severance Hospital from December 2019 to March 2020. We recruited 10 patients, two of whom were excluded from the trial because they had a history of other medical issues of screening failure. In total, the study included eight patients: six who underwent MRgFUS thalamotomy for ET and two who were subjected to MRgFUS pallidotomy for PD. Each patient provided informed consent, and the study was conducted with the permission and under the supervision of the Severance Hospital Institutional Review Board (Seoul, South Korea), and the Korea Food and Drug Administration (Figure 1). The clinical trial was registered at ClinicalTrials.gov (identifier number: NCT03935581). Each patient underwent CT to evaluate their SDR, which was calculated using the CT Density Analysis Tool (InSightec). Skull volume was measured using three-dimensional image software (Aquarius version 4.4; TeraRecon, Foster City, CA, United States).

### Autofocusing Echo Imaging Technique

**FIGURE 2:**
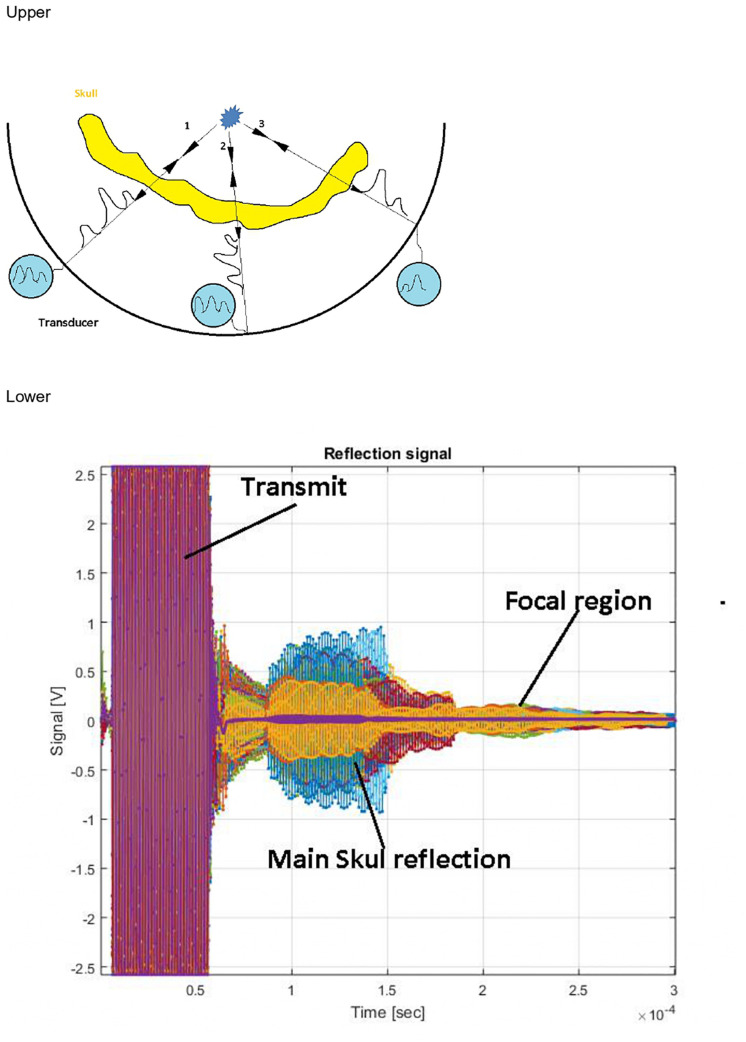
Upper: Ultrasound contrast agent microbubbles emit a spherical wave as a point scattering source. Aberration corrections are deduced directly from the measurement. Lower: Typical recorded signal. Different colors represent different channels. Multiple reflections from the skull dominate the signal. The microbubble signal is invisible at this scale.

The MRgFUS procedure was performed in a 3-T MRI scanner (GE Medical Systems, Milwaukee, WI, United States) using the Exablate 4000 device (InSightec), which has a 1,024-element phase array transducer that incorporates an independent phase and amplitude control for each element. The autofocusing echo imaging technique was applied (Figure 2). The clinical study was performed using the 650-kHz system, although the autofocusing echo imaging technique itself is not limited to a specific frequency.

Autofocusing echo imaging functions by introducing a point scatterer in the focal region. This point scatterer reflects ultrasound illumination in a spherical wave form, regardless of the incident wave properties. This wave reflected from inside the brain allows direct measurement of phase and amplitude distortions that can be used to optimize phase correction and relative amplitude calibration (Figure 2).

Like routine ultrasound imaging, to achieve imaging of microbubble activity with low intensity ultrasound waves, introduction of an Ultrasound Contrast Agent (USCA) is required (Qin et al., 2009). The USCA micro bubbles (DEFINITY^®^), with well-known and controlled size and concentration (0.08 μl/kg DEFINITY^®^ activated with Vialmix^®^ machine), are injected into the patient’s blood stream in bolus before the autofocusing echo imaging sequence is applied. Then, through an injection of the DEFINITY along with a transmission of a series of low-power, low-energy FUS pulses (The pulse power range for AF imaging is 7–300 vs. 100–1,500 W during actual treatment). The autofocusing echo imaging technique can be applied and used to improve the focusing quality. At the end of the autofocusing scan, the ExAblate Neuro Type 1.0 system will automatically calculate the phase and amplitude corrections necessary for the system to produce the sonication at the desired location and volume. Following the AF echo imaging scans, a T2^∗^/GRE MR sequence will be performed to evaluate any possible signs of hemorrhage. In addition, a rapid MR sequence will be performed to check for the presence of bleeding, edema, or any other adverse radiologic effect. This will be a safety check. In the event of new neurological deficits, other imaging modalities (including CT) will be performed immediately in addition to neurological and physical examination. The ExAblate system will not allow treatment to proceed for at least 12 min from the time of the last injection of DEFINITY to allow complete absorption of the microbubbles. Ultrasound contrast agent microbubbles are used as a reflector and are administrated into the blood stream. Although these microbubbles provide a strong signal, the emerging echo signal from the target region is still about 60 dB weaker than reflections from the surrounding skull, which strongly reflect the incident signal and heavily attenuate the signals from the microbubbles. Time-of-flight separation does not resolve this problem because there are multiple reflections, and the irregular skull base is close to the ultrasound source (Figure 2). Instead, because the microbubble signal has intrinsic non-linearity and stochasticity, it can be differentiated from the strong background signal by analyzing signal stability.

**FIGURE 3:**
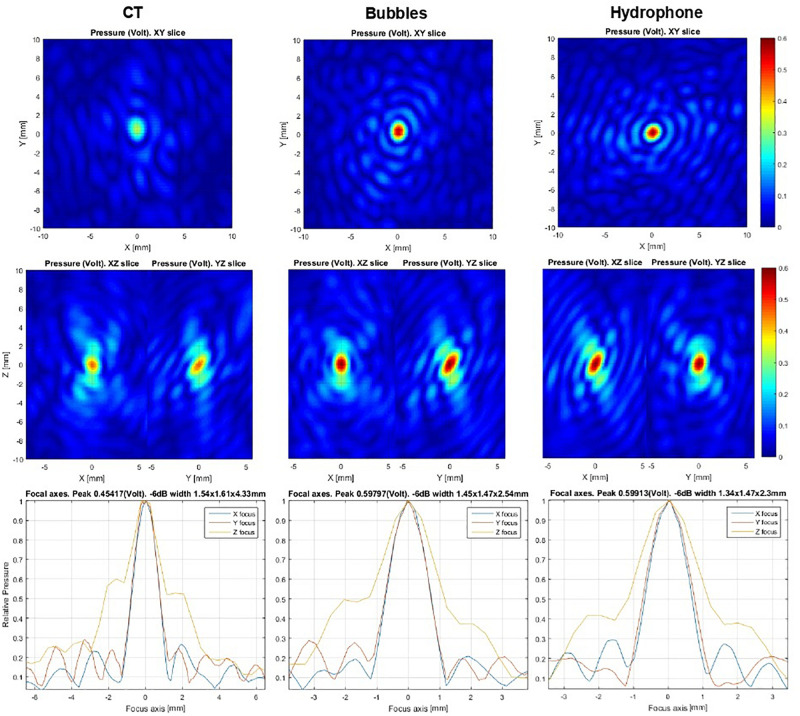
*Ex vivo* hydrophone focal scans at the 30 mm off-center point in the skull. The left column shows computed tomography-based correction used as an initial guess. The middle column represents microbubble-based correction, while the right column depicts a hydrophone-based result. The same acoustic power was emitted for all scans. Relative peak intensity values can be used as focusing accuracy criteria. Algorithm similarity score: AF/CT = 0.55, AF/hydrophone = 0.85.

For this reason, clinicians using this technique must implement simultaneous measurements on all channels. In the Exablate system, all power transmission channels are also used as acquisition channels. The acquisition hardware is a proprietary development of Insightec and consists of 32 acquisition cards, each of which consists of 32 channels. Each card has embedded computing and can be programmed independently for acquisition and signal processing. The programming and data exchange are performed through a local Ethernet network. To enhance the signal-to-noise ratio and dynamic range, I/Q demodulation was implemented in burst signals of ∼50 μs. These bursts (trigger frames) are repeated with variable periods of several microseconds each. The background and microbubble response signals are separated based on a search for short-lived stochastic events. An initial guess for phase correction is preferable to produce local field maxima near the desired target. No initial phase information or iterative approach is necessary for this method. The calculations of the echo imaging correction do not take into account the phase information of the initial guess. The microbubble concentration should be sufficiently low to allow time separation of single microbubble detection events. The acoustic power level should be sufficient to excite the microbubbles. Further increases in power lead to additional stochastic signals from living tissue that degrade signal quality. Pulse power is increased manually to ensure the patient’s safety. The number of detected microbubble signals is reported to the physician as a feedback about signal quality. Twenty detected events are typically enough to allow effective correction. Data collection stops automatically in the software at above 60 detected events. Ideally, each peak detected can be used to correct aberrations. However, signals are mixed and should be checked for validity. The algorithm models the signal as a spherical waveform centered at an arbitrary point. Phase front distortion is assumed to be the same for all point sources. Each candidate waveform is checked for correlation with the others to establish the data set to be used for aberration correction. The amount of correlations between peaks detected is reported to the physician as an “autofocusing score,” which is a value between 0.0 and 1.0 assigned to each acoustic correction table (ACT) produced from successful autofocusing sonication. The autofocusing score denotes the quality of the ACT. ACTs with an autofocusing score below 0.50 are discarded. For a given spot, the ACT with the highest score will be selected for use with the autofocusing echo imaging method. In addition, the result is compared to previously used corrections from echo imaging measurements or CT-based modeling and reported as a “similarity score” (0.0–1.0), which denotes the similarity between the newly calculated ACT and the ACT currently in use. Similarity is expected to be high (>0.8) between autofocusing ACTs. Lower values may indicate unreliable measurement and that additional autofocusing sonication should be performed. If there is no active autofocusing ACT for the current active spot, a CT-based ACT is used as a reference, although the similarity may be low. A given autofocusing ACT is only valid at the active spot for which it was calculated, allowing for location adjustments ≯5 mm and ideally <3 mm. The score values represent peak intensity ratios within the framework of the algorithm. The user must perform at least two independent echo imaging measurements to ensure that the results are consistent. Based on these measurements, the physician decides whether the correction is usable. As established by hydrophone scans (Figure 3) in a previous *ex vivo* study, echo imaging correction is similar to hydrophone-based correction, which is more sophisticated rather than CT-based scans. In addition to phase distortion correction, relative transmission can be extracted for each element in post-processing analysis. In future software versions, this will be used for precise *in situ* simulation of the focal spot shape.

### Clinical Evaluation

For patients with ET, for evaluating tremor, a maximum upper extremity clinical rating scale for tremor (CRST) sub-score of 32 points was calculated in the treated, tremor-dominant hand by summing the observed and performance-based scores from Parts A and B on the treated or contralateral side before and 1 month after MRgFUS thalamotomy. In patients with PD, symptoms were evaluated with the score of Part 3 of the unified PD rating scale (UPDRS), which was measured before and 1 month after MRgFUS pallidotomy.

### Data and Statistics

Statistics were performed using SPSS 25 (IBM Corporation, New York, NY, United States). Linear regression tests were measured to measure the relevancy of various factors, while Tmax measurement and the paired *t*-test were used to compare clinical improvement after the procedure. All *p*-values < 0.05 were considered statistically significant.

## Results

In total, eight patients underwent MRgFUS lesioning using the autofocusing echo imaging technique. Among these patients, six had ET and two had PD. The six patients with ET underwent left thalamotomy because all were right-handed and their baseline mean CRST score in the dominant hand was 34 (range: 27–53). The two patients with PD underwent left pallidotomy to control levodopa-induced dyskinesia; their baseline mean UPDRS Part 3 score was 36 (34 and 38).

The mean SDR of the patients with ET was 0.34 (range: 0.29–0.39), and the mean skull volume was 280.57 cm^3^. The mean SDR of the patients with PD was 0.41 (range: 0.38–0.44), and the mean skull volume was 287.13 cm^3^ (range: 271–303 cm^3^; Table 1).

**TABLE 1:** The demography of patients with magnetic resonance guided focused ultrasound surgery (MRgFUS) autofocusing (AF) echo imaging.

**Characteristics**	**Value**
Total patients	8
**Age**	
Mean (range)	68.87 (61–74)
**Diagnosis (target)**	
ET (VIM)	6
PD (GPi)	2
Mean SDR (total)	0.35
SDR (ET)	0.34
SDR (PD)	0.41
Mean volume (total)	282.21
Volume (ET)	280.57
Volume (PD)	287.13

*Note. ET, essential tremor; PD, Parkinson’s disease; VIM, Ventral intermediate nucleus; GPi: Globus pallidus interna; and SDR, skull density ratio.*

During the MRgFUS, an average of 15 sonications were performed, among which an average of 5.63 used the autofocusing echo imaging technique. The autofocusing echo imaging technique showed a higher similarity score, which means a higher reliability. The average similarity score between CT and AF was 0.58; in contrast, the average similarity score between multiple autofocusing technique was 0.95. The mean Tmax achieved was 55.88°C (range: 52–59°C). The mean energy delivered was 34.75 kJ (range: 20–42 kJ; Table 2).

**TABLE 2:** Summary of outcomes of MRgFUS AF echo imaging technique.

**Patient**	**Dx**	**Target**	**Volume**	**SDR**	**Sonication number**	**Energy delivered (KJ)**	**Tmax (°C)**	**AF sonication**	**AF power (W)**	**AF/CT similarity score**	**AF2/AF1 similarity score**	**Internal bubble score**	**Symptom**	**S/E**
1	ET	VIM, Left	291	0.37	15	33	58	9	75	0.57	0.89	0.89	Improved	None
2	ET	VIM, Left	227	0.39	16	20	59	4	25	0.63	0.99	0.85	Improved	None
3	ET	VIM, Left	290.97	0.36	12	35	57	3	20	0.68	0.99	0.91	Improved	None
4	ET	VIM, Left	319.24	0.31	12	39	55	5	25	0.61	0.97	0.87	Improved	None
5	ET	VIM, Left	299.23	0.29	19	42	56	4	27	0.7	0.96	0.81	Improved	None
6	PD	GPi, Left	271	0.38	13	40	52	9	85	0.34	0.97	0.79	Improved	None
7	ET	VIM, Left	256	0.33	16	37	57	4	30	0.68	0.89	0.88	Improved	None
8	PD	GPi, Left	303.26	0.44	17	32	53	7	70	0.43	0.95	0.81	Improved	None
Average			282.21	0.36	15	34.75	55.88	5.63	44.63	0.58	0.95	0.85		

*Note. Dx, Diagnosis; ET, essential tremor; PD, Parkinson’s disease; VIM, Ventral intermediate nucleus; GPi, Globus pallidus interna; SDR, skull density ratio; AF, auto focusing echo imaging; Tmax, Maximal temperature; and S/E, side effect.*

The mean post-MRgFUS lesioning CRST was 11 (range: 0–17), which was measured 1 month after the MRgFUS (Table 3). The dominant hand CRST score differed significantly 1 month after MRgFUS (*p* = 0.002 by paired *t*-test), while the score in the non-dominant hand did not (*p* = 0.530). In addition, performance score (CRST Part C) differed significantly after MRgFUS (*p* = 0.003). No serious adverse events occurred during or after treatment. Among the sonication factors (skull volume, SDR, sonication number, autofocusing score, similarity score, energy range and power, and Tmax), none differed significantly (*p* > 0.05), and the autofocusing score showed a *p*-value of 0.071 (Table 2).

**TABLE 3:** Comparison of pre and post MRgFUS AF echo imaging thalamotomy changes of CRST score for ET patients.

**Patient**	**Pre op**			**Post op**		
	**Right**	**Left**	**Part C**	**Right**	**Left**	**Part C**
1	17	12	12	5	11	3
2	13	16	18	0	17	0
3	28	26	25	8	27	7
4	18	11	14	10	13	7
5	16	10	11	11	7	5
6	19	7	15	6	11	1
Mean	18.5	13.7	15.8	6.7	14.3	3.8

After the MRgFUS lesioning, the UPDRS score showed significant improvement. The mean UPDRS Part 3 score of the two patients with PD was reduced from 36 to 24 (26 and 22), 1 month after the MRgFUS autofocusing echo imaging pallidotomy. However, because of the small number of patients, we could not measure statistical significance. No adverse effects of microbubbles were observed in all eight patients. No serious adverse events occurred during or after MRgFUS lesioning using the autofocusing echo imaging technique.

## Discussion

Although MRgFUS is safe and efficient, the number of patients indicated for the procedure is limited because the appropriate treatment temperature cannot be reached in some patients. Specifically, patients with a low SDR are typically excluded from MRgFUS treatment (Chang et al., 2016, 2019; Jung et al., 2019). We previously reported the importance of measuring SDR and the clinical significance of SDR scores (Chang et al., 2016, 2019; Jung et al., 2019). The SDR indicates the ratio of Hounsfield unit value between the marrow and cortical bone on the CT (Chang et al., 2016). A low SDR indicates that the cortical bone has a higher density than the trabecular bone (Chang et al., 2016). When ultrasound waves pass through the skull, they refract, reflect, and attenuate because of differences in acoustic impedance.

In studies of SDR in the normal population, one third of the people had an SDR below 0.4, which is generally considered unfavorable for MRgFUS in ET. In patients with PD, the target GPi is more lateral than the VIM, indicating a more diverse incident angle. This is also an obstacle for MRgFUS, resulting in significant energy loss, distortion of the penetrating ultrasound beam, and unsuccessful lesioning (Jung et al., 2019). Thus, only patients with an SDR above 0.45 are indicated for MRgFUS pallidotomy to treat PD.

**FIGURE 4:**
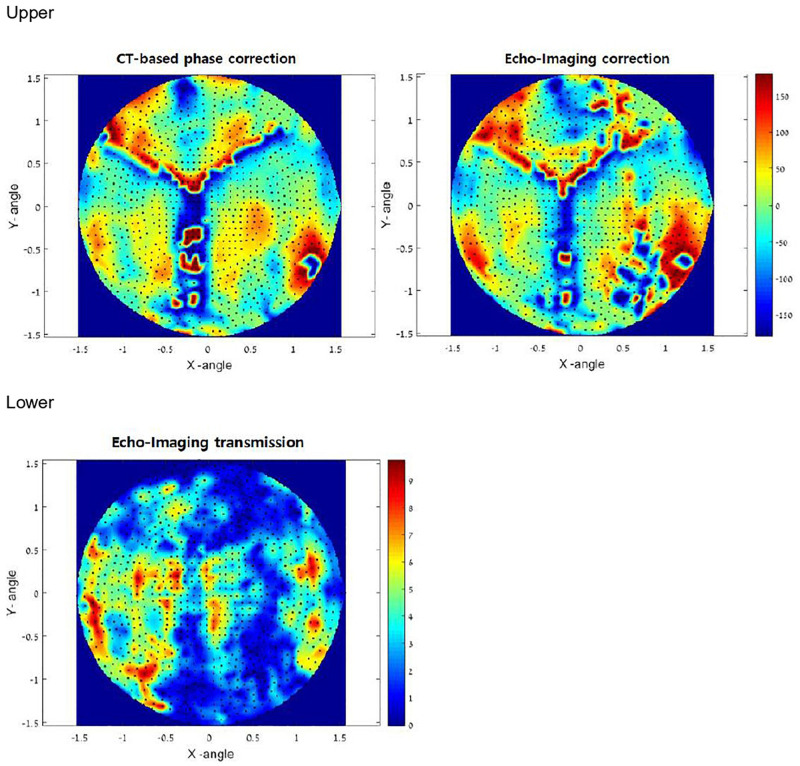
Upper: *In vivo* phase correction compared in a clinical experiment. The left side shows computed tomography (CT)-based correction. The right side represents the echo imaging method. The image is projected onto the transducer surface. Echo imaging correction is calculated independently of the CT-based phases. Algorithm similarity score: AF/CT = 0.63. Lower: Transmission signal amplitudes (relative) measured *in vivo* using echo imaging. Image is projected onto the transducer surface.

To treat targets in the brain through the intact skull, clinicians must refocus the beam after it passes through the non-uniform, non-spherical bony tissue of the skull. Focus correction predicts skull-generated phase aberrations at each target point inside the brain and corrects the phases of each transducer element to compensate for it. Until now, focus correction was based on an acoustic model derived from high-resolution pre-operative CT images of the skull. The autofocusing technique was developed to overcome the ultrasound characteristics such as distortion and reflections. The autofocusing echo imaging technique used in the present study relies on accurate measurement of the ultrasound echo by each of the 1,024 transducer elements. This data can then be used to compute the phase delay per element. Autofocusing echo imaging in the present study allowed more accurate correction than previous CT-based correction (Figure 4).

**FIGURE 5:**
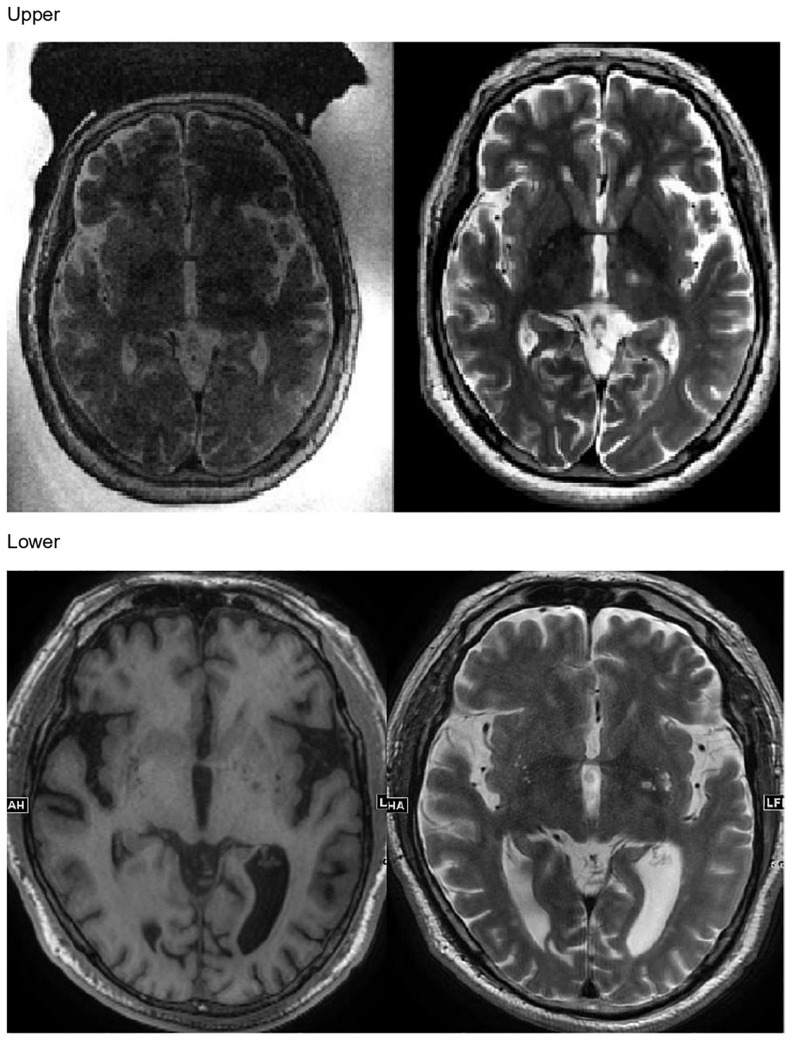
Upper: Magnetic resonance (MR) images taken immediately after MR-guided focused ultrasound autofocusing echo imaging thalamotomy in a patient with essential tremor. Lower: MR images taken immediately after MR-guided focused ultrasound autofocusing echo imaging pallidotomy in a patient with Parkinson’s disease.

In the present study, every patient successfully underwent MRgFUS lesioning using the autofocusing echo imaging technique without any side effects. None of the patients had headache or nausea during the procedure. The target lesions were made exactly in the planned area (VIM thalamus for patients with ET and GPi for patients with PD), as confirmed by follow-up MRI in all patients (Figure 5). Immediately after the sonication, the patients’ clinical symptoms were subjectively improved, as were the CRST score in patients with ET and the UPDRS score in patients with PD.

Temperature was over 54°C in all patients with ET. In those with PD, the Tmax was over 52°C. We could have raised the Tmax to 54°C in these patients by raising the energy, but the targeted ablative lesion was confirmed by MRI, so no additional sonication was carried out to avoid side effects. Since the GPi is more lateral than the VIM and therefore requires a different ultrasound incident angle, it was difficult to raise the temperature of the targeting lesion using the previous MRgFUS method. However, using the autofocusing echo imaging technique, we raised the target temperature enough for lesioning.

With the autofocusing echo imaging technique, patients with ET who have an SDR below 0.4 and those with PD who have an SDR below 0.45 could achieve the ideal therapeutic temperature to create a permanent lesion, even though MRgFUS was previously contraindicated in these patients.

Although the present study was conducted using a small number of patients, it showed the efficacy of both MRgFUS thalamotomy and pallidotomy using the autofocusing echo imaging technique. Furthermore, it showed that this technique can even be used in patients with an SDR below 0.4, unlike previous methods.

Recently, various techniques to overcome SDR, skull volume, and incident angle have been used in the clinical field to overcome such barriers, such as increasing the sonication number or sonication power. However, these techniques show adverse effects, such as failure of thermal lesioning, necrosis in the skull bone marrow, abnormal thermal shape of the target, or abnormal cavitation signal in an unexpected lesion (Schwartz et al., 2018; Wang et al., 2018).

Further research with larger populations and follow-ups is required to confirm the efficacy and unexpected side effects of MRgFUS lesioning using autofocusing echo imaging. Using the new autofocusing echo imaging technique, complications of the previous MRgFUS lesioning would be reduced. Autofocusing echo imaging has focusing ability similar to hydrophone-based methods. It produces sharp and tight spots with higher peak intensity and temperature. Clinicians must account for these new parameters when applying the technique. For example, treatment strategy based on the peak temperature will lead to smaller lesions than previously.

## Conclusion

Using the autofocusing echo imaging technique, MRgFUS lesioning treatment is now safe and efficient in patients with low SDR. As such, the technique could expand the indications for MRgFUS lesioning to include patients with ET patients who have an SDR of <0.4 and patients with PD who have an SDR < 0.45.

## Data Availability Statement

The original contributions presented in the study are included in the article/supplementary material, further inquiries can be directed to the corresponding author/s.

## Ethics Statement

The studies involving human participants were reviewed and approved by Severance hospital Institutional Review Board (IRB). The patients/participants provided their written informed consent to participate in this study.

## Author Contributions

KWC collected clinical data, analyzed the data, and wrote this manuscript. WSC and HHJ collected clinical data. IR, EZ, and OP helped with interpretation of the results, edited the manuscript. JWC designed the study, helped with interpretation of the data, and edited the manuscript. All authors contributed to the article and approved the submitted version.

## Conflict of Interest

IR, EZ, and OP was employed by company InSightec. The authors declare that the research was conducted in the absence of any commercial or financial relationships that could be construed as a potential conflict of interest.
